# Supplementary material for: Impact of the 21-Gene Assay in Patients with High-Clinical Risk ER-Positive and HER2-Negative Early Breast Cancer: Results of the KARMA Dx Study
Source: Cancers (Basel). 2023 Feb 28;15(5):1529. doi: 10.3390/cancers15051529 (PMC10001004; doi:10.3390/cancers15051529)
Supplement: Supplementary file 1 [file cancers-15-01529-s001.zip › cancers-2135756-supplementary.pdf]

Supplementary Materials

Supplement A: Post hoc Analysis of Cohorts A and C, by TAILORx Recurrence Score Cutpoints

Background

Results of TAILORx showed that patients with ER+, HER2-negative, node-negative breast cancer and RS 0-25 had no benefit of adjuvant chemotherapy.[1,2] An exploratory analysis suggested that patients in TAILORx ≤50 years of age with RS 16-25 might have some chemotherapy benefit (Table S1).[2]

Table S1. Chemotherapy benefit by TAILORx Recurrence Score groups and age.

Recurrence Score Group	Age Group	Chemotherapy Benefit
RS 0-25	>50 years of age	<1%
RS 0-15	≤50 years of age	<1%
RS 16-20	≤50 years of age	~1.6% benefit
RS 21-25	≤50 years of age	~6.5% benefit
RS 26-100	All ages	>15% benefit

Methods

Here we undertook a post hoc analysis of the KARMA Dx patients with node-negative or micrometastatic breast cancer to estimate the chemotherapy benefit that might be expected if treatment decisions were based on TAILORx Recurrence Score cutpoints.

Results

A total of 59 node-negative patients from Cohorts A and C were included in this analysis. Stratification of patients by TAILORx Recurrence Score groups and age is shown in Table S2.

Table S2. Number (%) of node-negative patients in KARMA Dx Cohorts A and C (combined), by TAILORx Recurrence Score group and age.

Recurrence Score Group	Age Group	Chemotherapy Benefit	No. of Patients	No. (%) of Patients that change to omit CT
RS 0-25	>50 years of age	<1%	24	20 (83%)
RS 0-15	≤50 years of age	<1%	8	6 (75%)
RS 16-20	≤50 years of age	~1.6% benefit	7	3 (43%)
RS 21-25	≤50 years of age	~6.5% benefit	3	1 (33%)
RS 26-100	All ages	>15% benefit	17	1 (6%)

Conclusions

Simulating treatment decisions based on TAILORx Recurrence Score groups showed that 39 (66%) patients had a Recurrence Score result associated with little or no chemotherapy benefit, 3 (5%) patients with some chemotherapy benefit, and 17 (29%) with substantial chemotherapy benefit.

Supplement B: The 21-gene Oncotype DX Breast Recurrence Score®.

Table S3: List of 21 genes analyzed in the KARMA Dx study

Group	Genes
Proliferation	Ki67; ST15; Survivin; CCNB1 (Cyclin B1); MYBL2
Invasion	MMP11 (Stromolysin 3); CTSL2 (Cathepsin L2)
Her2	GRB2; HER2
Estrogen	ER; PGR; BCL2; SCUBE2
Other	GSTM1; CD68; BAG1
Reference genes	ACTB (b-actin); GADPH; RPLPO; GUS; TFRC

Figure S1: Final gene panel and to design an algorithm to compute a Recurrence Score (RS)

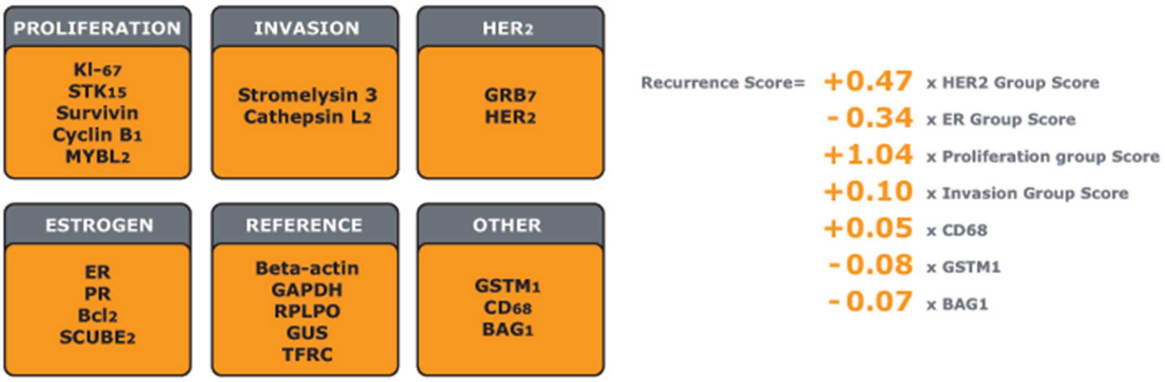

For the development of the 21-gene Oncotype DX Breast Recurrence Score® a list of 250 candidate genes was initially selected from the published literature, genomic databases, pathway analysis, and from microarray-based gene expression profiling.

Three independent breast cancer clinical studies were performed in a total of 447 (patients to test the relationship between the expression of the 250 candidate genes and disease recurrence. The results from these three preliminary studies were used to select the final gene panel and to design an algorithm to compute a Recurrence Score (RS) (1-3).

The RS was then validated in the large homogeneous populations of the NSABP B-14 and NSABP B-20 studies, which provided the test with initial evidence for clinical utility. Shown below are the forest plots of the Kaplan-Meier estimate for the Hazard Ratios for all 16 cancer-related genes in both studies, with the predictive power of genes group being greater than that of individual genes (Figure S2).

Figure S2: The forest plots of the Kaplan-Meier estimate for the Hazard Ratios for all 16 cancer-related genes

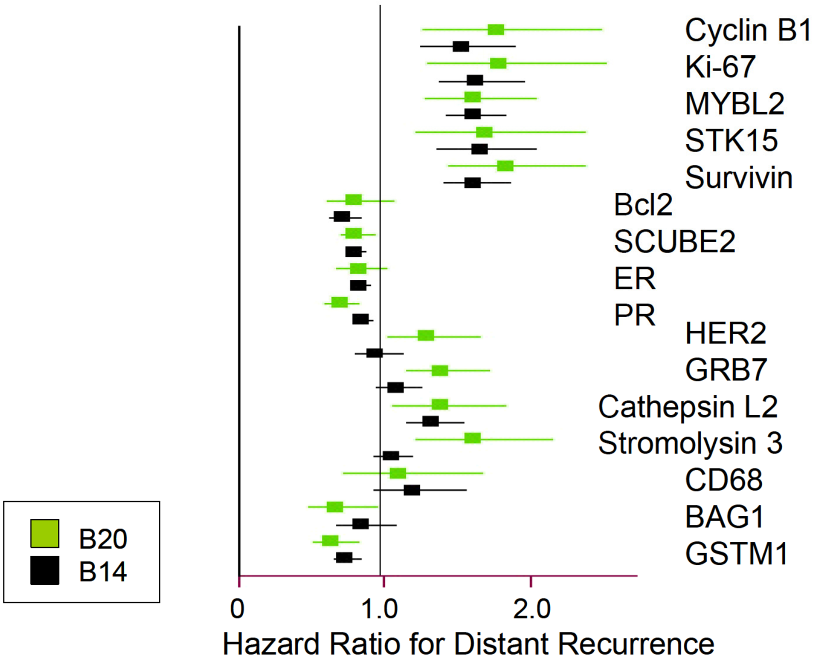

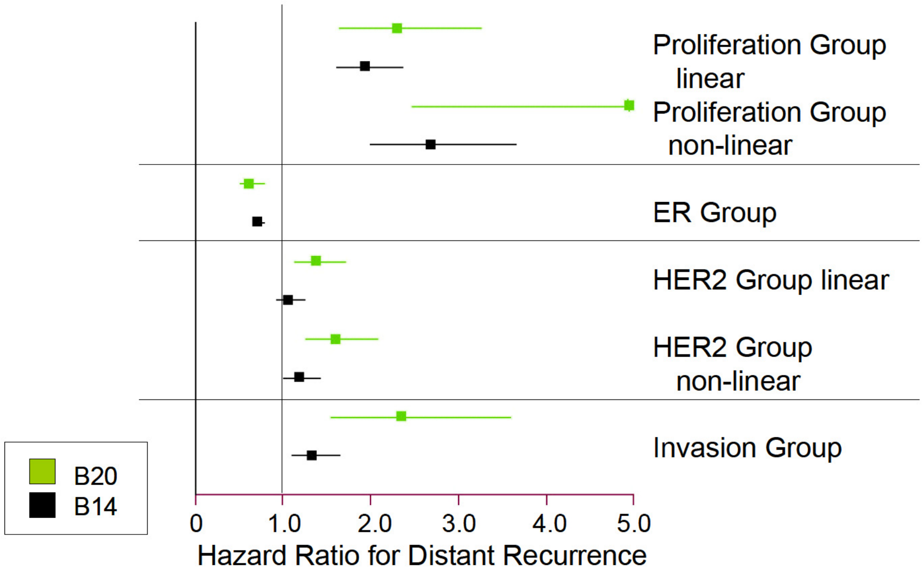

References:

1. Paik, S.; Tang, G.; Shak, S.; Kim, C.; Baker, J.; Kim, W.; Cronin, M.; Baehner, F.L.; Watson, D.; Bryant, J.; Costantino, J.P.; Geyer CEJr Wickerham, D.L.; Wolmark, N. Gene expression and benefit of chemotherapy in women with node-negative, estrogen receptor-positive breast cancer. J Clin Oncol. **2006**, *24*, 3726–3734. doi: 10.1200/JCO.2005.04.7985.
2. Cobleigh, M.A.; Tabesh, B.; Bitterman, P.; Baker, J.; Cronin, M.; Liu, M.L.; Borchik, R.; Mosquera, J.M.; Walker, M.G.; Shak, S. Tumor gene expression and prognosis in breast cancer patients with 10 or more positive lymph nodes. Clin Cancer Res. **2005,** *11*, 8623–8631. doi: 10.1158/1078-0432.CCR-05-0735.
3. Esteban, J.; Baker, J.; Liu, M.L.; MGLlamas, M.G.; Walker, M.G.; Mena, R. Tumor gene expression and prognosis in breast cancer: Multi-gene RT-PCR assay of paraffin-embedded tissue. Proc. Am. Soc. Clin. Oncol, **2003**. https://www.scienceopen.com/document?vid=f5fe5404-4d43-4ac6-acb0-b04ebc37d14d (Accessed on 17 December 2022)
